# Reconstruction of Type I-II Internal Hemipelvectomy in a Patient With Pelvic Myxoid Chondrosarcoma: A Case Report

**DOI:** 10.7759/cureus.26621

**Published:** 2022-07-06

**Authors:** Pablo Escudero-Acurio, Francisco Mahaluf, Luis Bahamonde

**Affiliations:** 1 Escuela de Medicina, Universidad San Sebastián, Concepcion, CHL; 2 Hip Team, Traumatology, Dr. Víctor Ríos Ruiz Healthcare Complex, Los Ángeles, CHL; 3 Orthopaedic Department, Universidad de Chile, Santiago, CHL

**Keywords:** chondrosarcoma, hemipelvectomy, pelvic reconstruction surgery, custom-made prosthesis, case report

## Abstract

Pelvic chondrosarcomas are a major clinical challenge since the only therapeutic approach available is surgical resection. Reconstruction after partial resection of the pelvis including the acetabulum or the hip joint is a laborious and rigorous surgical procedure. Numerous complications are associated with different reparative methods. Moreover, due to the anatomical complexities of the area, adequate surgical margins are difficult to achieve in many cases, which are closely related to the advent of local recurrence of the tumor. Several techniques for hip function restoration and skeletal reconstruction have been reported. The purpose of this report is to describe a novel pelvic reconstruction technique for PI-II resection that required a custom-designed implant. We present the case of a 61-year-old female patient with chronic pain in the gluteal region. The pelvis’s magnetic resonance imaging (MRI) showed an osteolytic tumor in the right iliac wing that compromises the acetabular roof. The diagnosis was a grade 2 central chondrosarcoma. Surgery included the reconstruction of the acetabulum by inserting two Schanz pins coated with hydroxyapatite, one in the iliopubic corridor and the other in the ischium. A supporting “pyramid” was built, unitizing both Schanz with cement, onto which an acetabular cage was inserted. The procedure was completed with a conventional total hip prosthesis. The patient presented an acute prosthesis infection, which positively responded to prompt surgical lavage and antibiotic treatment. After 10 months of follow-up, the patient remains free of infection, with weight-bearing as tolerated, without pain, and with excellent hip motion. No tumor recurrence has occurred. Medialization of the construct has occurred as expected, with no evidence of implant loosening. The technique used in this patient is novel, could be considered cost-effective, and has allowed the reconstruction of a functional hip. For resections of the acetabular area and preservation of the ischium and pelvic zones, this technique may be an acceptable option.

## Introduction

Bone tumors of the pelvis represent 10%-15% of primary malignant bone tumors [1]. Chondrosarcoma is the third most common, after myeloma and osteosarcoma, accounting for one of five malignant bone tumors [2]. Chondrosarcoma is a tumor producing a non-osteoid cartilage matrix by neoplastic cells [3]. It usually affects adults between 40 and 70 years old, with the pelvis being the most frequent location, and is often present in men [1,2,4].

Pelvic chondrosarcomas represent a complex clinical and surgical problem. This is because these tumors are radioresistant and chemoresistant, and the only therapeutic approach available is surgical resection. Therefore, the intervention should proceed in a way that margins are kept free of tumor tissue, which poses an additional difficulty [5].

For tumors located in the acetabular region (type II resections, according to Enneking classification) [5], internal hemipelvectomy (preserving the lower extremity) is the procedure of choice. External or classical hemipelvectomy is a radical amputation, indicated only for selected cases, in which conservative techniques are impossible, or when the remnant limb would result nonfunctional, due to the loss of at least two of the three following elements: hip joint, femoral nerve, and sciatic nerve [1,4]. Internal hemipelvectomy, if approached along with reconstructive techniques for the hip, is very challenging as well. It has the goal of functional restoration and consequent better functional results, although there is no clear evidence of its advantages versus non-reconstruction [1,6].

With advances in implantation techniques, oncology surgeons have gradually adopted prosthetic reconstruction of the pelvis after tumor resection [7]. Reconstructive options can prevent significant differences in leg length and preserve hip joint function [5]. Complications are frequent, however, including infection, prosthesis loosening and failure, and fractures of the remaining bone or allografts, when used [5,7].

The choice of a particular technique for hip joint reconstruction depends on prognosis, functional capabilities of the patient, and particular surgical scenario and type of resection. A systematic review analyzed the clinical outcomes of internal hemipelvectomy, comparing up to seven types of reconstruction techniques, with unique challenges and complications for each method [8]. Moreover, only case reports or case series are usually reported, due to the rarity of the disease [1,2].

This manuscript describes a pelvic reconstruction technique after a type I-II internal hemipelvectomy, for which hydroxyapatite Schanz pins, cement, and an acetabular cage were used.

## Case presentation

We present a case of a 61-year-old female patient with a history of hypothyroidism and obesity and several months of pain in the right hip and buttock.

**Figure 1:**
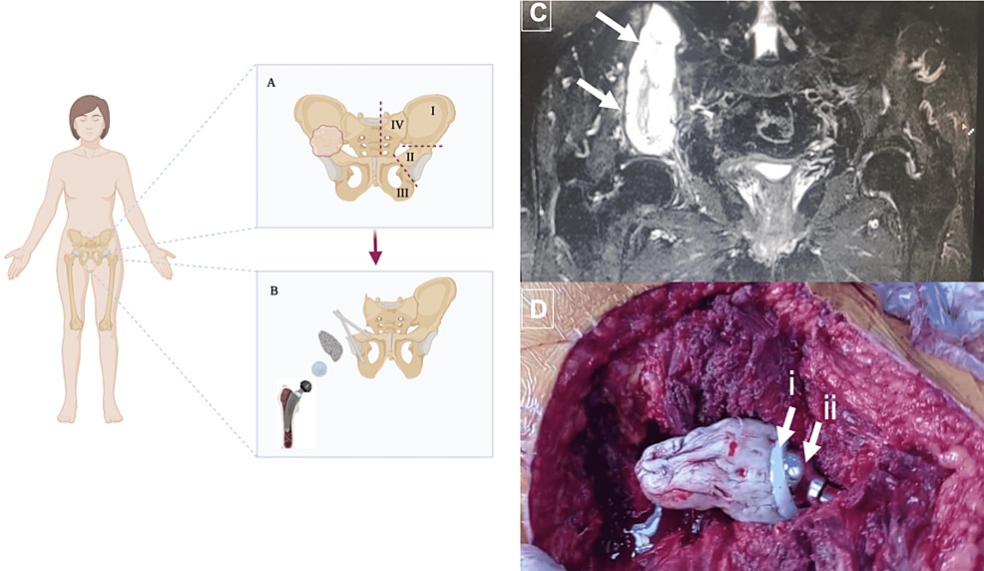
Reconstruction of hemipelvectomy I-II. (A) The right pelvis illustrating the localization of low-grade myxoid chondrosarcoma in the iliac wing and acetabular roof. The left pelvis showing the classification of hemipelvectomies according to Enneking and Dunham [5]. (B) Illustration of surgical technique with custom-made hydroxyapatite Schanz prosthesis and total hip prosthesis. (C) Preoperative pelvic MRI showing a large heterogeneous osteolytic tumor (arrows) in the right iliac wing that compromises the acetabular roof. (D) (i) Cemented pyramid in the hydroxyapatite Schanz, where an acetabular reconstruction basket and hip replacement were implanted, and (ii) an uncemented 36-mm head stem.

Magnetic resonance imaging (MRI) of the pelvis showed an osteolytic tumor in the right iliac wing that compromised the acetabular roof, corresponding to the Enneking and Dunham PI-II classification (Figure 1). Computed tomography was negative for metastasis in the chest, abdomen, and pelvis. The Hospital Oncology Committee discussed the case.

A surgical biopsy of the lesion reported low-grade myxoid chondrosarcoma. After reanalysis by the Hospital Oncology Committee, an internal hemipelvectomy and hip reconstruction were planned.

Surgical technique

An extensive ilioinguinal approach was performed. The resection included the right hemipelvis, from the cephalic acetabular half to the sacroiliac joint, leaving only the caudal half of the acetabulum in continuity with the pubic rami. Two hydroxyapatite-coated Schanz pins were inserted, one in the iliopubic corridor and the other in the ischium. A cemented pyramid was molded by unitizing both Schanz, with cement, in which an acetabular reconstruction cage (Stryker, Kalamazoo, Michigan, USA) was implanted, and a 46-mm cemented plastic insert was placed (Figure 1).

An uncemented stem with a 36-mm head was placed in the femur, achieving a stable hip. The cemented “pyramid,” as a consequence of weight-bearing, was assumed to be finally supported by the sacral ala due to progressive medialization.

Postoperative deep infection occurred, with the presence of *Staphylococcus aureus*, *Enterobacter cloacae*, and *Enterococcus faecalis*. This was managed by prompt surgical debridement and implant retention, with the addition of intravenous (IV) antibiotics vancomycin + imipenem. Six weeks of additional oral treatment was continued with ciprofloxacin + amoxicillin-clavulanic acid. After the completion of treatment, the surgical wound removed has persisted without fistulae nor discharge and with nearly normal laboratory parameters.

**Figure 2:**
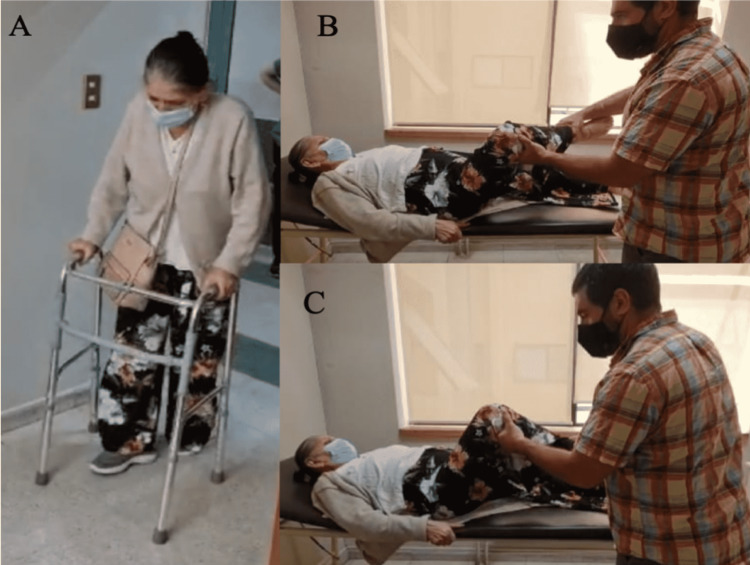
Limb functionality. (A) The patient maintains a supported gait with slight external rotation. (B) Full range in external rotation to passive mobilization (0º-35º). (C) Hip flexion on passive mobilization (0º-120º).

**Figure 3:**
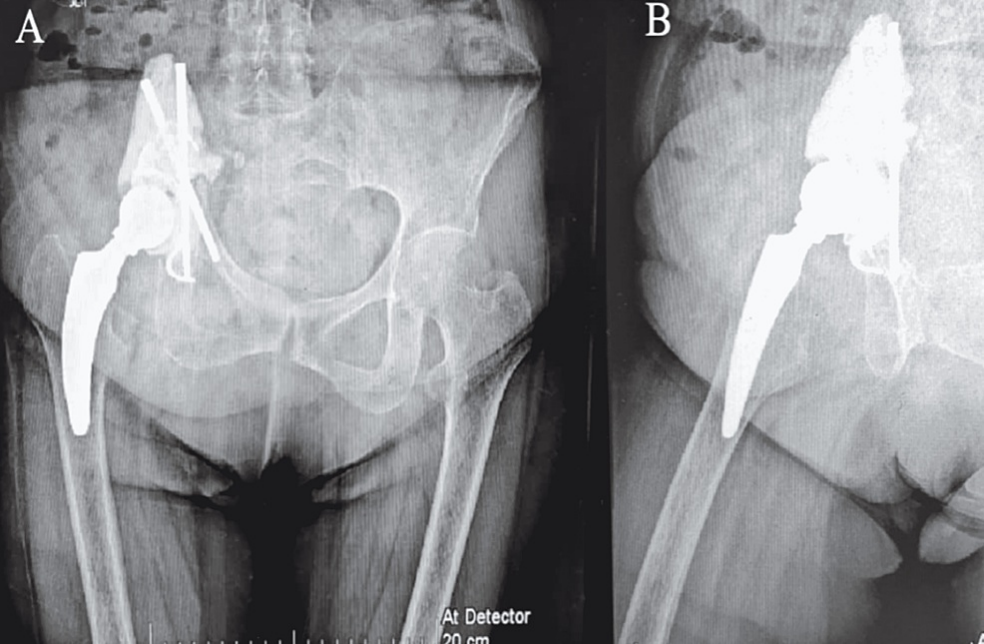
Postoperative X-ray at seven months of control. (A and B) Anteroposterior views in neutral and abduction showing a fixed prosthesis, with medialization and vertical final position of the cup.

After a 10-month follow-up, she was in good general condition, with a healed and healthy wound, and walking without pain with assistance (Figure 2). X-ray analysis showed that the prosthesis remains stable, with medialization of the joint as expected and no additional complications (Figure 3). To date, there has been no tumor recurrence.

## Discussion

Pelvic chondrosarcomas pose significant challenges for management, since only surgical resection, attempting tumor-free margins, remains the treatment of choice. The anatomical complexity of the pelvis increases the difficulties of surgery, as well as the numerous complications associated with attempts at functional reconstruction, especially in cases of acetabular resections (type II resections, according to Enneking and Dunham classification) [5]. Diverse subtypes of chondrosarcomas are known. An epidemiological analysis of the American database of bone tumors between 1973 and 2013 found 687 patients with myxoid chondrosarcomas, such as the case we present, occurring predominantly in males, over 50 years old, and with a five-year survival rate of close to 50%, due to metastatic spread [3].

Clinically, our patient presented dull and constant pain, as most patients with this diagnosis [2,3]. Radicular-type pain, sensory or motor disturbances, deep vein thrombosis (DVT), claudication, or changes in bowel function are suggestive of advanced disease due to the direct infiltration or extrinsic compression of the tumor [1].

Chemotherapy and radiotherapy do not have a role as effective adjuvants against chondroid tumors [2,3,5]. External hemipelvectomy (radical limb amputation) was the gold standard for many years due to the complex anatomy of the pelvis, the extent of many tumors at the moment of diagnosis, and the difficulty in reconstructing major bone defects [2]. However, with the development of imaging, for better analysis of the location and extent of the tumor, and improvements in surgical techniques, limb-sparing procedures have emerged as a viable surgical option [4]. The patient we presented precisely accounted for this type of tumor resection, which sought to preserve the limb, improving the patient’s functionality.

Pelvic tumors may affect more than one of the regions described by Enneking [5,9]. If the ilium is intact (type II + III resection), it may be used as support for endoprostheses, such as the “ice cream cone”-like [10] or the saddle-like prostheses [11]. The possible alternative reconstruction options are hip arthrodesis or pseudoarthrodesis [12]. In their classic forms, these procedures require the presence of an iliac remnant to achieve iliofemoral fusion. In addition, a modified method has been reported that consists of raising the femoral head to the lateral side of the sacrum and confining it with a pseudocapsule made with trevira (pseudoarthrosis) [13]. Unfortunately, this procedure has a high reoperation rate (40%) due to limb shortening, necrosis, or femoral head displacement. Also, patients have the risk of deep infection (one out of every three operated). These adverse events affect clinical outcomes in recovering/maintaining the hip function, reaching only 62% in the functionality score of the Musculoskeletal Tumor Society (MSTS) test after this particular surgery [13]. Another alternative is a reconstruction based on pelvic allografts or a combination of allografts and prostheses. A systematic review that included nine studies with 133 patients in which this technique was used [8] concluded that the mean MSTS score was 72%, and deep infection was the main complication (15% of cases). Despite this systematic evidence, there are also reports of a functional MSTS score of 53.3% and complications due to deep infection in up to 35% of patients with type PI-II and PI-II-III resections [14]. Similarly, patients treated with extracorporeal irradiated tumor autograft and total hip replacement for type PI-II resections had a success rate of 55% [15] and an MSTS functional score for all types of resections of 72% [8].

A novel reconstruction technique for bone defects in patients with periacetabular tumors is the manufacturing of 3D-printed (customized) prostheses. A mean MSTS score of 80% functionality and no postsurgical complications have been reported in a series [16]. It is a field of growing interest among surgeons due to the precise matching of the implant to a particular bone defect and the addition of customized fixation to the remnant bone specific to the patient. A systematic review analyzing five studies involving 182 patients has reported a mean MSTS functionality score of 63% in patients using personalized prostheses [8]. Despite these results, it must be considered that there are only a few reported cases with type PI-II resections and the use of custom implants. Regarding complications with this method, 23% have been deep infections [8]. However, this complication is common for the vast majority of reconstructive techniques. Causes are associated with large tumor size, anatomical complexity (and therefore the duration of the surgery), and the use of massive implants and/or allograft bone. These complications add uncertainty regarding the patient’s outcomes [4,5,9].

The case we presented corresponds to type PI-II resection, where the ilium and the proximal part and roof of the acetabulum were removed. As such, there is no remnant bone for cranial support of a device. The technique designed for reconstruction considered a custom-made prosthesis with hydroxyapatite Schanz, cement, and cage. The aim, such as with other methods, was to restore a functional and stable hip joint. The construct was designed to progressively medialize with weight-bearing, till supporting against the sacral ala. Of note is the fact that medialization led to a more vertical position of the cup, originally inserted at 40° of inclination. Being this a matter of concern for the fate of the construct, should this method be indicated in future cases, care must be taken to provide a nearly horizontal orientation for the cup. This may lead to a more favorable inclination angle when medialization is completed.

The main limitation of the case presented here is the short-term follow-up. Complications such as tumor recurrence and/or mechanical failure, and infection are still possible.

However, in the case of type II and I + II resections, it can be a valuable, low-cost, and cost-effective option, as it does not require sacrificing the limb and allows for satisfactory functional recovery of the hip joint in the short term. Utilization of the remaining bone corridors for hydroxyapatite-coated Schanz fixation, unitizing them with cement, in which a cage can be inserted, along with progressive support of the construct against the sacral ala, could be validated as an alternative to more costly methods. Allografts, allografts + prosthesis composites, and customized implants have been more extensively utilized. Nonetheless, complications that may lead to failure are still frequent with any of these. We see no reason indicating that the outcome associated with our method may be less favorable.

## Conclusions

In summary, we present a patient with low-grade myxoid chondrosarcoma that compromised the right iliac wing and acetabular roof (type I + II resection) who underwent a reconstructive surgical technique that preserved the limb and allowed a satisfactory functional recovery of the hip joint in the short term. The method used in this patient may be cost-effective, if, when evaluated in more patients and with longer follow-up, functional results and complications prove at least comparable to others.
